# Atomic‐Scale Investigation of the Lattice‐Asymmetry‐Driven Anisotropic Sublimation in GaN

**DOI:** 10.1002/advs.202106028

**Published:** 2022-06-02

**Authors:** Shanshan Sheng, Tao Wang, Shangfeng Liu, Fang Liu, Bowen Sheng, Ye Yuan, Duo Li, Zhaoying Chen, Renchun Tao, Ling Chen, Baoqing Zhang, Jiajia Yang, Ping Wang, Ding Wang, Xiaoxiao Sun, Jingmin Zhang, Jun Xu, Weikun Ge, Bo Shen, Xinqiang Wang

**Affiliations:** ^1^ State Key Laboratory for Mesoscopic Physics and Frontiers Science Center for Nano‐optoelectronics School of Physics Peking University Beijing 100871 China; ^2^ Electron Microscopy Laboratory School of Physics Peking University Beijing 100871 China; ^3^ Songshan Lake Materials Laboratory Dongguan Guangdong 523808 China; ^4^ Collaborative Innovation Center of Quantum Matter Peking University Beijing 100871 China; ^5^ Peking University Yangtze Delta Institute of Optoelectronics Nantong Jiangsu 226010 China

**Keywords:** anisotropic sublimation, equilibrium crystal structure, in situ transmission electron microscope, wurtzite gallium nitride

## Abstract

Thermal sublimation, a specific method to fabricate semiconductor nanowires, is an effective way to understand growth behavior as well. Utilizing a high‐resolution transmission electron microscope (TEM) with in situ heating capability, the lattice‐asymmetry‐driven anisotropic sublimation behavior is demonstrated of wurtzite GaN: sublimation preferentially occurs along the [$000\bar{1}$] and [0001] directions in both GaN thin films and nanowires. Hexagonal pyramidal nanostructures consisting of six semipolar $\left\{1\bar{1}01\right\}$ planes and one (000$\bar{1}$) plane with the apex pointing to the [0001] direction are generated as a sublimation‐induced equilibrium crystal structure, which is consistent with the lattice‐asymmetry‐driven growth behaviors in wurtzite GaN. These findings offer a new insight into the thermal stability of wurtzite GaN and provide essential background for tailoring the structure of III‐nitrides for atomic‐scale manufacturing.

## Graphical abstract

Anisotropic sublimation behavior of wurtzite GaN is directly demonstrated using in situ heating transmission electron microscopy (TEM) technique. Sublimation preferentially occurs along [0001] and [$000\bar{1}$] directions. A hexagonal pyramid with the apex pointing to [0001] direction is generated as the sublimation‐induced equilibrium crystal structure of wurtzite GaN. The observed phenomena is attributed to the asymmetric surface energies in wurtzite GaN.

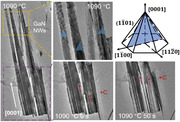

## 1 Introduction

Nanostructures based on III‐nitrides are of great interest because of their promising applications in high‐power electronic devices, optoelectronic devices, quantum information devices, etc.^[^ ^1^, ^2^, ^3^, ^4^ ^]^ Today, difficulties in the precise control of their position, geometry, and structure pose serious challenges for the design and manufacture of III‐nitride‐based nanodevices. III‐nitride nanowires, nanotripods, nanotubes, nanowalls, and horn structures have been fabricated using a variety of methods, including spontaneous growth,^[^ ^5^ ^]^ thermal sublimation,^[^ ^6^ ^]^ selective area growth,^[^ ^7^ ^]^ patterning, and etching,^[^ ^8^ ^]^ or a combination of these processes.^[^ ^9^ ^]^ Among them, thermal sublimation offers better control over site and size, introduces much less contamination and damage to sidewalls, and is compatible with spontaneous growth. Thus, thermal sublimation is an attractive method to manufacture of III‐nitride‐based nanostructures and nanodevices at the atomic scale.^[^ ^10^, ^11^, ^12^, ^13^ ^]^ In addition, as a reverse process of growth, thermal sublimation provides additional information for the analysis of surface stability and motion. Therefore, the analysis of sublimation not only provides essential understanding of the fabrication of nanostructure by thermal annealing, but is also of paramount importance in predicting stable crystal planes during growth and sublimation.

The behavior of wurtzite GaN at elevated temperatures has been studied using various measurement methods such as Raman scattering,^[^ ^14^ ^]^ laser reflection,^[^ ^15^ ^]^ and reflection high‐energy electron diffraction (RHEED), etc.^[^ ^13^, ^16^, ^17^ ^]^ However, details such as structural evolution, sublimation mechanisms, and the role of defects during sublimation remain unclear. Recent advancements of in situ heating transmission electron microscopy (TEM) enable us to directly observe and analyze sublimation kinetics with atomic resolution in real time, which were previously inaccessible by conventional measurement methods.

In this study, we used a TEM equipped with a heating stage to investigate the sublimation behavior of wurtzite GaN at elevated temperatures. Here, for the first time, the real‐time structural evolution of GaN film and nanowires is observed, which directly reveals the anisotropic sublimation behavior of wurtzite GaN. The prevalence of the difference in surface energies due to the asymmetry of the lattice during heating was established. Finally, a sublimation‐induced hexagonal pyramid was generated as an equilibrium crystal structure of wurtzite GaN with a size lower than tens of nanometers. This provides a fundamental basis for controlling the morphology of III‐nitrides at the atomic scale to develop advanced structures and devices.

## 2 Results and Discussions

**Figure 1:**
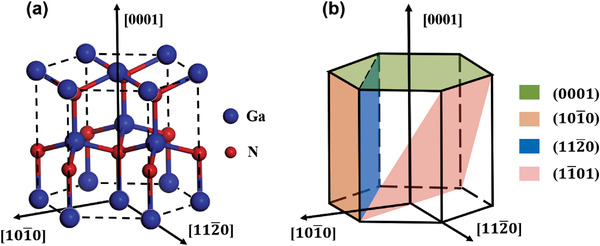
Wurtzite crystal structure of a) GaN and b) representation of the most relevant low‐index crystal planes.

Unlike most of conventional III–V semiconductor compounds such as GaAs or InP which crystallize in the zinc‐blende phase, GaN exhibits wurtzite structure. **Figure** **1**a shows the unit cell of wurtzite GaN. Due to inversion symmetry center broken and deviation from an ideal tetrahedral coordination along the [0001] axis, wurtzite GaN has an anisotropic crystal structure and a nonzero spontaneous polarization along ‐*c* axis. Different planes include polar planes, nonpolar planes, and semipolar planes are defined as shown in Figure 1b. The fundamental properties of these planes are also anisotropic, such as phonon anisotropy^[^ ^18^, ^19^ ^]^ and anisotropic thermal expansion.^[^ ^20^ ^]^

As one of basic factors in surface physics, surface energy plays an important role in faceting, crystal growth, and equilibrium crystal shape. For wurtzite structures, it is possible to calculate surface energies of symmetric nonpolar surfaces by standard slab methods, such as *m*‐ and *a*‐surfaces. However, it is difficult to obtain energies of polar or semipolar surfaces because of the absence of symmetry.^[^ ^24^ ^]^ Moreover, polar and semipolar surfaces contain different types and numbers of dangling bonds, which results in their surface energies being strongly dependent on chemical potential of the environment.^[^ ^22^ ^]^ Although the exact individual energy values for polar and semipolar planes are not yet available, based on these studies, the relative energy with respect to other planes under various chemical potential conditions can be obtained. This offers some understanding of the equilibrium morphologies and growth or sublimation rate in the direction normal to each plane. Under the real growth conditions of nanowire (N‐rich condition) in molecular beam epitaxy (MBE), the surface energies of the nonpolar ($1\bar{1}00$) m‐ and ($11\bar{2}0$) *a*‐planes are rather lower than those of other planes.^[^ ^21^ ^]^ Therefore, the sidewalls of nanowires grown along c axis are usually *m*‐ or *a*‐plane.^[^ ^25^ ^]^ During the sublimation of GaN, the instant surface is believed to be covered by Ga monolayer since it is more stable than that covered by N monolayer. **Table** **1** shows the calculated surface energies of typical planes of wurtzite GaN under Ga‐rich conditions. Whereas low index semipolar planes and ($000\bar{1}$) plane have lower energy than other planes. In this way, a special anisotropic sublimation behavior will occur at elevated temperature as a result of differences in surface energies for different crystal orientations.

**Table 1:** Calculated surface energies (meVÅ^−2^) of typical planes of wurtzite GaN. The calculations are performed using chemical potentials for Ga‐rich environment.

Method	(0001)	($000\bar{1}$)	($1\bar{1}00$)	($11\bar{2}0$)	($1\bar{1}01$)
DFT^[^ ^21^ ^]^	≈150	98	76	120	87
DFT^[^ ^22^ ^]^	≈136	≈78	≈98	≈103	≈100
DFT^[^ ^23^ ^]^	≈167	≈87	≈118	≈122	≈110

**Figure 2:**
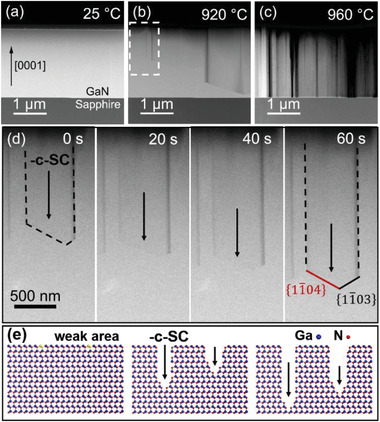
Cross‐sectional HAADF‐STEM images of a GaN film at different temperatures taken along the $\left[11\bar{2}0\right]$ direction: a) 25 °C, b) 7 min at 920 °C, c) 3 min at 960 °C. d) Sublimation process over time at 920 °C at the area marked with a white box in (b), showing a sublimation channel (SC) toward the ‐*c* direction. The inclined facets of V‐tip are $\left\{1\bar{1}04\right\}$ and $\left\{1\bar{1}03\right\}$, respectively. e) Atomic model of different stages of sublimation.

A Ga‐polar GaN lamella was loaded into TEM chamber and an in situ TEM heating experiment was conducted to investigate the sublimation behavior of the GaN film. The thickness of the lamella is ≈100 nm. **Figure** **2**a–c shows high‐angle annular dark‐field scanning transmission electron microscopy (HAADF‐STEM) images of the GaN film in the temperature range 920–960 °C. Figure S4 (Supporting Information) shows a plot of the temperature against heating time. At temperature up to 900 °C, the GaN lamella showed no noticeable change. When the temperature reaches 920 ℃, the GaN sublimates from the (0001) plane, followed by many sublimation channels in the ‐*c* direction, as shown in Figure 2d. The sublimation channels have a V‐shaped tip instead of a flat tip (Figure 2d). The left inclined facet of V‐shaped tip corresponds to $\left\{1\bar{1}04\right\}$ planes and the right inclined facet of V‐shaped tip corresponds to $\left\{1\bar{1}03\right\}$ planes. This phenomenon is consistent with the fact that, upon thermal sublimation in the molecular beam epitaxy (MBE) chamber under high vacuum, 3D islands with sixfold symmetry are formed on the surface of the GaN film.^[^ ^13^, ^17^ ^]^

We think that the unique sublimation channel along ‐*c* direction is attributed to the anisotropic sublimation rates of different planes of GaN. First, the sublimation starts from weak bonding sites on (0001) surface, such as lattice defects. With heating, different planes, including the *c*‐plane, *m*‐plane and semipolar planes sublimate simultaneously. Fast‐sublimating *c*‐facets gradually shrink while slow‐sublimating *m*‐ and semi‐ facets expand and ultimately define the channel shape. Then, vertical sublimation channels along ‐*c* direction form due to the lowest sublimation rate of m‐planes (Figure 2d). According to the real‐time evolution of sublimation channel (Figure 2d), the sublimation rate along ‐*c* direction is estimated to be about 9 nm s^−1^ which is higher than previously reported values in vacuum.^[^ ^26^, ^27^ ^]^ That is due to the higher sublimation temperature used in our experiment. The channel length and width were measured along with the heating time and the ratio of sublimation rates between ‐*c* and *m* directions was found to be ≈40:1. These results suggest that the (0001) plane has a much higher surface energy, and therefore, lower thermal stability than other planes, favoring sublimation toward the ‐*c* direction. A similar phenomenon was also observed in GaN films during etching in H_2_ at high temperatures, where the *c*‐axis is the dominant etching direction.^[^ ^28^, ^29^ ^]^ Our results provide direct evidence of the anisotropic thermal stability of different planes in wurtzite GaN. After the heating process, nanopillars were formed along the *c*‐axis as a result of strong sublimation toward the ‐*c* direction, as shown in Figure 2c. The formation of nanopillars during heating is similar to the selective area sublimation (SAS) process.^[^ ^6^ ^]^ In the SAS approach, well‐controlled nanowires along the *c*‐axis were obtained using a SiN*_x_* mask on GaN‐based epitaxial layers during thermal sublimation. In our experiment, wherein a mask was not employed during thermal sublimation, the channels originated from weak bonding sites on surface and sublimated along the ‐*c* direction. Therefore, the obtained nanopillars along the *c*‐axis were irregular, as shown in Figure 2c.

**Figure 3:**
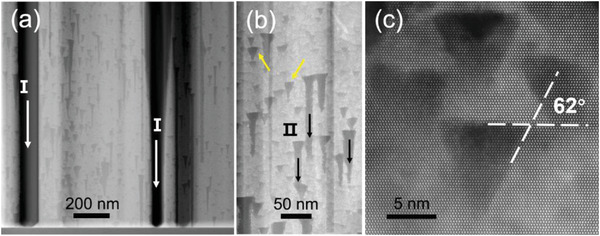
High‐resolution HAADF‐STEM images of GaN lamella taken along the $\left[11\bar{2}0\right]$ direction. The dark area is the sublimated area and the light area is the reserved area. a) Image of a selective area of GaN lamella after an in situ TEM heating process. Sublimation channels emerge from the surface marked as I. b) Image of GaN film at a high magnification, showing multiple triangular voids, marked with yellow arrows and new sublimation channels originating from triangular voids, marked as II. c) Depiction of triangular voids at an atomic scale.

To investigate the structural evolution after the heating process, the temperature was lowered to room temperature for high‐resolution measurements. As shown in **Figure** **3**a, the sublimation channels (marked I) shown in Figure 2 stopped at the interface between GaN and sapphire. In addition to sublimation channels I, many triangular voids appeared in the GaN lamella (marked with yellow arrows, Figure 3b), and those new sublimation channels originated from the triangular voids (marked II, Figure 3b). The triangular voids may originate from weak bonding sites on the sidewall of GaN lamella at high temperatures, such as lattice defects. A high‐resolution HAADF‐STEM image of the triangular voids (Figure 3c) shows that the angle between the inclined facets of the triangular side and the (0001) plane is 62°. The details of this semipolar plane are discussed below. Once these voids appear, many new sublimation channels are formed, marked by sign II in Figure 3b, as an extension of these voids along the ‐*c* direction, because the new space of the (0001) plane has been exposed in a vacuum. These sublimation channels are also mainly decomposed toward the ‐*c* direction. Generally, sublimation occurs more easily in area with weak bonding, such as dislocation sites.^[^ ^30^ ^]^ However, in our experiment, dislocations were not observed near the sublimation channels, and the density of the sublimation channels calculated from Figure S3 (Supporting Information) is ≈1.89 × 10^10^ cm^−2^. This value is higher than the dislocation density of the GaN film grown by metal organic chemical vapor deposition (MOCVD), which is ≈ 5 × 10^8^ cm^−2^. Therefore, we deduce that sublimation channels are the products of intrinsic sublimation. Furthermore, self‐assembled nanowires have a very low dislocation density owing to stress relaxation during growth. The TEM images of sublimation channels shown in Figure 5 also support our conclusion.

**Figure 4:**
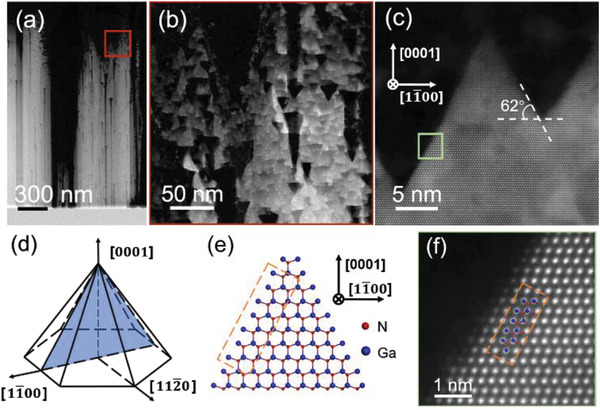
HAADF‐STEM images of GaN lamella with atomic resolution, taken along the $\left[11\bar{2}0\right]$ direction: a) Image of GaN lamella after the heating process. b) Image of triangles obtained from a selective area in (a). c) Edge of the triangular shaped structure. The angle between the inclined facets and the (0001) plane is 62°. d) Structure of a hexagonal pyramid consisting of six $\left\{1\bar{1}01\right\}$ planes and one (000$\bar{1}$) plane, and the triangular shape represents when viewed from the [$11\bar{2}$0] direction. e) Atomic structure of the triangular shape. f) Atomic arrangement of Ga and N atoms. The structure of the triangular shape is in good agreement with the atomic arrangement of Ga and N atoms.

**Figure** **4**a shows the HAADF‐STEM images of the GaN film after the heating process. The zoom in area marked as red rectangular is shown in Figure 4b. It is shown that, instead of sublimation channels along the *c*‐axis, interesting structures consisting of many triangles, pointing to the (0001) direction, emerged near the surface. High‐resolution images of the triangles (Figure 4c) show that the angle between the inclined facets and the (0001) facet is also 62°. This corresponds to the angle between the inclined facets and the (0001) facet of the triangular voids mentioned above (Figure 3c). These results suggest that the inclined facet corresponds to one of the $\left\{1\bar{1}01\right\}$ planes, because the angle between the $\left\{1\bar{1}01\right\}$ planes and (0001) plane is also 62°.^[^ ^31^ ^]^ Hexagonal pyramids formed by six well‐defined {1$\bar{1}01$} planes were also observed during GaN growth.^[^ ^32^ ^]^ The pyramid projected onto the plane normal to the [$11\bar{2}0$] direction has a triangular shape, as shown in Figure 4d. Furthermore, the arrangement of atoms in the ($1\bar{1}01$) planes, schematically depicted in Figure 4e, perfectly matches our HAADF‐STEM image (Figure 4f). Pyramid consisting of {$1\bar{1}01$} planes at the top was predicted as an equilibrium crystal shape using first principles and ab‐initio approach.^[^ ^24^, ^33^ ^]^ The theoretical prediction that the Ga‐terminated $\left(1\bar{1}01\right)$ surface has the lowest energy under Ga‐rich conditions, which is also in good agreement with our results. Thus, we believe that the triangular shape is actually a hexagonal pyramid composed of six of $\left\{1\bar{1}01\right\}$ planes and one (000$\bar{1}$) plane as a base in the 3D space. This specific sublimation behavior at the edge of GaN lamella can be explained by sublimation starting from the *m*‐planes with lattice defects. As shown in Figure 4a, the sidewalls of the empty channels induced by sublimation along ‐*c* direction are *m*‐planes. The lattice defects that generated during growth on exposed *m*‐planes provide more sites for sublimation. Because the sublimation rates of semipolar $\left\{1\bar{1}01\right\}$ planes are lower than *m*‐planes, $\left\{1\bar{1}01\right\}$ planes are retained during the sublimation process, resulting in the formation of hexagonal pyramids consisting of six $\left\{1\bar{1}01\right\}$ planes.

**Figure 5:**
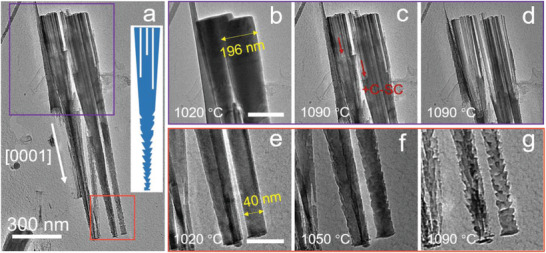
Bright field TEM images of the evolution of GaN nanowires under different heating conditions. a) TEM image of GaN nanowires at 1090 °C. b–d) Magnified images of the area marked with purple box in (a) under different heating conditions (1020 °C 55 s, 1090 °C 0 s, and 1090 °C 50 s), showing sublimation channels toward the *c* direction at the top half of the nanowires. e–g) Magnified images of the area marked with orange box in (a) under different heating conditions (1020 °C 55 s, 1050 °C 60 s, and 1090 °C 50 s), showing a triangular sublimation behavior at the bottom half of nanowires. The scale bar in (b) and (e) is 200 and 80 nm, respectively.

To better understand the universal sublimation behavior of GaN, in situ TEM heating experiment was also performed on self‐assembled GaN nanowires since nanowires have a very low defect density. The nanowires were grown by radio frequency plasma‐assisted MBE on a Si (111) substrate at 700 °C under a nitrogen‐rich condition. For self‐assembled GaN nanowires on Si (111), nitrogen polarity is usually maintained.^[^ ^34^ ^]^ The diameters of the nanowires gradually increased because of the lateral growth of the nanowire during the epitaxial process. As shown in **Figure** **5**b,e, the diameter at the top of the nanowire is ≈196 nm, but 40 nm at the bottom of the nanowire. Compared with the GaN film, nanowires serve as a better platform for investigating the behavior of GaN sublimation, as both the sidewall and surface are exposed to vacuum. A plot of temperature versus heating time for the nanowires sample is shown in Figure S4 (Supporting Information). Figure 5a shows a TEM image of the nanowires at 1090 °C. The inset in Figure 5a shows a schematic model of sublimation behavior of the nanowires. Similar to the GaN lamella, sublimation channels are formed along the *c* direction in the upper half of the nanowires (Figure 5b–d). However, the direction of propagation of sublimation channels in nanowires is opposite to that of the sublimation channels in the GaN film, which means that the (000$\bar{1}$) plane is the preferred sublimation plane. In addition, the temperature at which observable sublimation occurs in nanowires is much higher than that in the GaN lamella (1090 ℃ for GaN nanowire versus 920 ℃ for GaN lamella). The higher sublimation temperature here probably reflects that the (000$\bar{1}$) plane has a better thermal stability than the (0001) plane.^[^ ^35^ ^]^ The sublimation rate of (000$\bar{1}$) plane is about 5 nm s^−1^ at 1090 °C. In addition, the tip of the sublimation channels toward *c* direction is flat, as shown in Figure 5f, which is different from the V‐shaped tip of the sublimation channels towards ‐*c* direction in Figure 2d. This phenomenon is consistent with the morphology of the nanowires tips during growth, where Ga‐polar nanowires tend to have a pencil‐like tip, while N‐polar nanowires tend to be flat.^[^ ^34^ ^]^ Interestingly, as the heating process continued, the sublimation channels along the *c* direction appeared only in the upper half of the nanowires. At the bottom of the nanowires, sublimation starts from the sidewall and the structures consist many triangles stacked together with the apices of the triangles pointing in the (0001) direction (Figure 5g). This inconsistent sublimation behavior can be interpreted by the lattice defects induced nonuniform sublimation process. At the top side of nanowires, sublimation starts from the regions with lattice defects on $\left(000\bar{1}\right)$ plane that generated with growth, propagated along [0001] direction. At the bottom side of nanowires, the exposed (0001) plane is almost free of defects due to the limited diameter (about 40 nm).^[^ ^36^ ^]^ Therefore, the sublimation prefers to start from the weak bonding sites on the sidewall of nanowires. Sublimation channels along the *c*‐axis grew rapidly with heating time (Figure 5b–d). In contrast, pyramidal‐shaped structures at the bottom of the GaN nanowires are stable at a heating temperature of 1090 °C (Figure 5g). Atomic resolution images of the triangles in the nanowires could not be obtained owing to the difficulty in adjusting the orientation of nanowires along the electron beam. Comparing these results with the triangular structures observed in GaN lamella (Figure 4b), we deduce that triangles in the nanowires have the same structure as triangles in the GaN lamella. As shown above, in both GaN films and GaN nanowires, hexagonal pyramids consisting of six $\left\{1\bar{1}01\right\}$ planes and one (000$\bar{1}$) plane form as an equilibrium crystal shape in GaN at high temperatures. This result can be used to verify theoretical calculations, which show that low‐index semipolar surfaces have the lowest surface energy.^[^ ^22^, ^24^ ^]^

## 3 Conclusion

In summary, we directly observed the GaN sublimation process using an advanced in situ TEM heating technique, and analyzed the sublimation behaviors. We show that the unique sublimation channels along [0001] and [000$\bar{1}$] direction is the preferred sublimation behavior in wurtzite GaN. Our results also reveal that this sublimation process highly depends on the lattice defects density on surface: the larger ones exhibit multichannel sublimation, while the smaller ones show a sidewall dominated sublimation. Finally, a hexagonal pyramid with the apex pointing in the [0001] direction consisting of six semipolar $\left\{1\bar{1}01\right\}$ planes and one (000$\bar{1}$) plane is proposed as the sublimation‐induced equilibrium crystal structure of wurtzite GaN. We attributed the observed phenomena to the asymmetric surface energies in wurtzite GaN. Our findings provide a fresh perspective on the design and application of GaN‐based nanodevices.

## 4 Experimental Section

### Growth

The GaN sample was prepared on sapphire substrates by metal organic chemical vapor deposition (MOCVD), using trimethylgallium (TMGa) and ammonia (NH_3_) as the Ga and N precursors, respectively. Then, GaN epitaxy was performed at a growth temperature of 1070 ℃ and a chamber pressure of 200 Torr. The precursor flows of TMGa and NH_3_ were set to 150 sccm and 48 slm, respectively. The thickness of the GaN layer is 2 µm with a growth rate of 3.5 µm h^−1^. The GaN nanowires sample was grown on a Si (111) substrate using PA‐MBE. The substrate was chemically cleaned with HF acid solution to remove oxides before loading it into the MBE chamber. The Si wafer was degassed under ultrahigh vacuum at 900 °C for 30 min. Subsequently, the nanowires was grown under nitrogen‐rich conditions, maintaining a nitrogen flow rate of 1.5 sccm. GaN nanowires with a height of ≈1.4 µm were grown at 700 °C.

### Sample Fabrication

The GaN film sample for in situ heating experiments was prepared by focused ion beam (FIB) — ThermoFisher Helios G4 UX. Further details of the fabrication process are presented in Figure S1 in the Supporting Information. The GaN nanowires for in situ heating process were dispersed in an alcohol solution and dropped onto a heating chip (Protochips).

### Characterization

For in situ TEM experiments, a commercial TEM holder (Protochips) allowing heating up to 1200 °C was applied. Transmission electron microscopy was performed using an FEI Tecnai F20 high‐resolution TEM at an accelerating voltage of 200 kV. STEM was performed using an FEI Titan cubed Themis G2 300 ultrahigh‐resolution TEM at an accelerating voltage of 300 kV. The heating was operated under a high vacuum of ≈4 × 10^−6^ Pa.

## Conflict of Interest

The authors declare no conflict of interest.

## Supporting information

Supporting Information

Supplemental Video 1

Supplemental Video 2

## Data Availability

The data that support the findings of this study are available on request from the corresponding author. The data are not publicly available due to privacy or ethical restrictions.
