# Supplementary material for: Atomic‐Scale Investigation of the Lattice‐Asymmetry‐Driven Anisotropic Sublimation in GaN
Source: Adv Sci (Weinh). 2022 Jun 2;9(22):2106028. doi: 10.1002/advs.202106028 (PMC9353495; doi:10.1002/advs.202106028)
Supplement: Supplementary file 1 — Supporting Information [file ADVS-9-2106028-s001.pdf]

ADVANCED SCIENCE Open Access

Supporting Information

for *Adv. Sci.*, DOI 10.1002/advs.202106028

Atomic-Scale Investigation of the Lattice-Asymmetry-Driven Anisotropic Sublimation in GaN

Shanshan Sheng, Tao Wang, Shangfeng Liu, Fang Liu, Bowen Sheng, Ye Yuan, Duo Li, Zhaoying Chen, Renchun Tao, Ling Chen, Baoqing Zhang, Jiajia Yang, Ping Wang, Ding Wang, Xiaoxiao Sun, Jingmin Zhang, Jun Xu, Weikun Ge, Bo Shen and Xinqiang Wang**

Supporting Information

Atomic-Scale Investigation of the Lattice-Asymmetry-Driven Anisotropic Sublimation in GaN

Shanshan Sheng, Tao Wang, Shangfeng Liu, Fang Liu, Bowen Sheng, Duo Li, Zhaoying Chen, Ling Chen, Jiajia Yang, Ping Wang, Ding Wang, Xiaoxiao Sun, Jingmin Zhang, Jun Xu, Weikun Ge, Bo Shen and Xinqiang Wang*.*

Supporting Movies S1. An in situ STEM movie showing the propagation of sublimation channel towards -c direction in GaN film at 920 °C.

Supporting Movies S2. An in situ TEM movie showing the sublimation process of GaN nanowires at 1050 °C. The diameter of nanowires are below 50 nm.

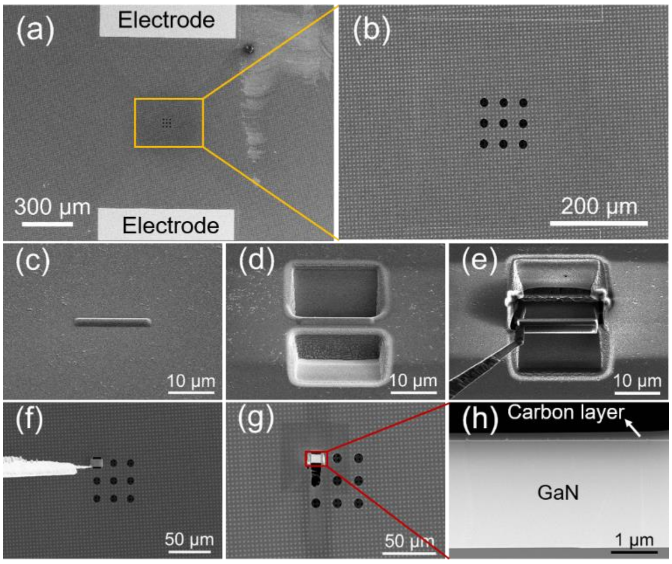

Figure S1. SEM image of Protochips (a) with nine observation windows (b) for in situ heating experiments. The series SEM images (c-g) show the procedure of specimen fabricated by FIB technique, including (c) covering with a protective carbon layer, (d) ion beam milling around selected area, (e) lift out a lamella, (f) attach

lamella to Protochips and (g) FIB cutting and milling. (h) The HAADF-STEM image of GaN lamella.

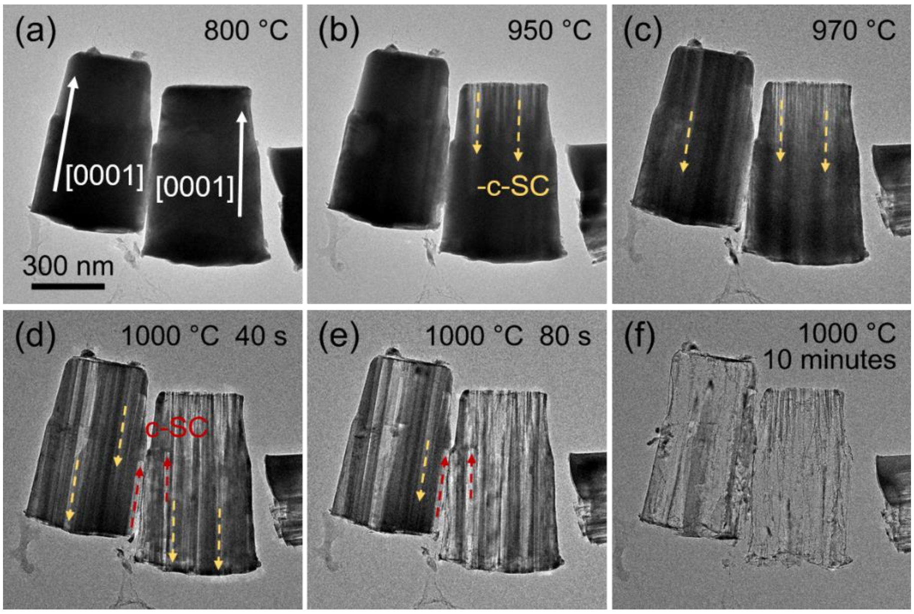

Figure S2. Bright filed TEM images of the evolution of GaN nanorods at different temperatures. The major sublimation channels (SC) are marked with the yellow and red arrows, indicating the -c and c directions, respectively.

As shown in **Figure S1**h, a carbon layer exits on the surface after the whole sample fabrication process. Fortunately, that carbon layer does not influence the sublimation behavior investigated in our experiment. In situ TEM heating experiment were also performed on GaN nanorods with diameter about 500 nm, where the GaN nanorods are free of carbon coverage. **Figure S2** shows the sublimation process of GaN nanorods. It is shown that sublimation channels start from the surface and propagate along c axis. This confirms that the carbon layer does not influence the sublimation behavior of GaN.

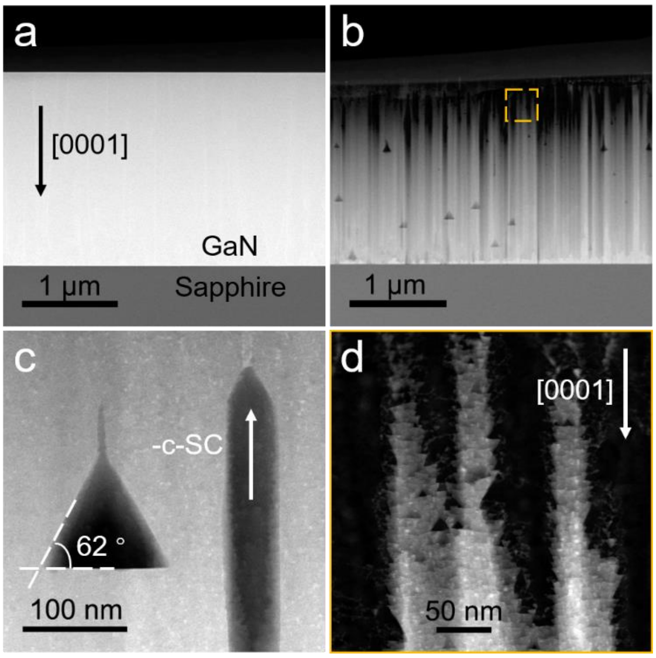

Figure S3. Cross-sectional HAADF-STEM images of N-polar GaN film on sapphire substrate taken along $[11\bar{2}0]$ direction: (a) 25 °C, (b) 1020 °C 14 min, (c) high magnification image of triangular void and sublimation channel inside GaN film, (d) high-resolution image of selected area in (b).

Figure S3 shows images of structural evolution of a N-polar film under in situ heating process. The triangular shape voids pointing to $[000\bar{1}]$ direction appear during the heating process (Figure S3b), and the angle between inclined facet and (0001) plane is 62°. The triangular shape structure pointing to [0001] directions is also reserved near the surface of GaN lamella as shown in Figure S3d. These results are in accordance with our proposed model in Figure 3 and Figure 4.

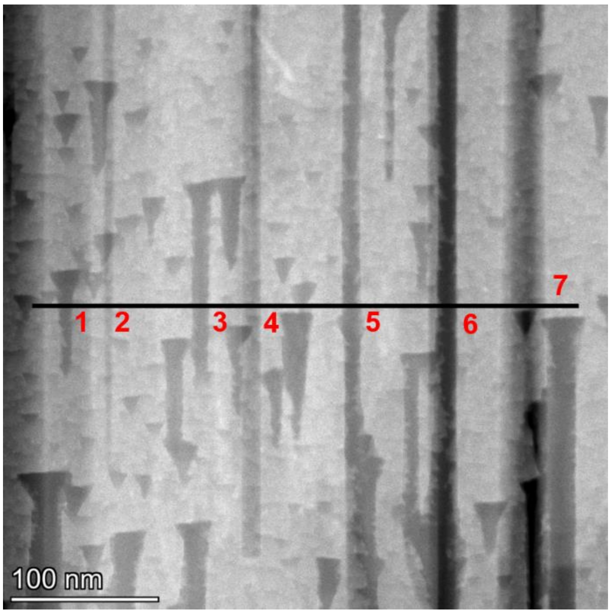

Figure S4. HAADF-STEM image of GaN lamella after the heating process. The dark area is the sublimated area and the light area is reserved area. The thickness (h) of this lamella is about 100 nm. The length of black line is 370 nm.

The density (ρ) of sublimation channel is calculated by formula

$$\rho = \frac{n}{Lh}$$

where L is the length of the line in Figure S4, n is the number of sublimation channel crossing the line, and h is the thickness of lamella. Thus, the density is about $1.89 \times 10^{10}\ cm^{-2}$.

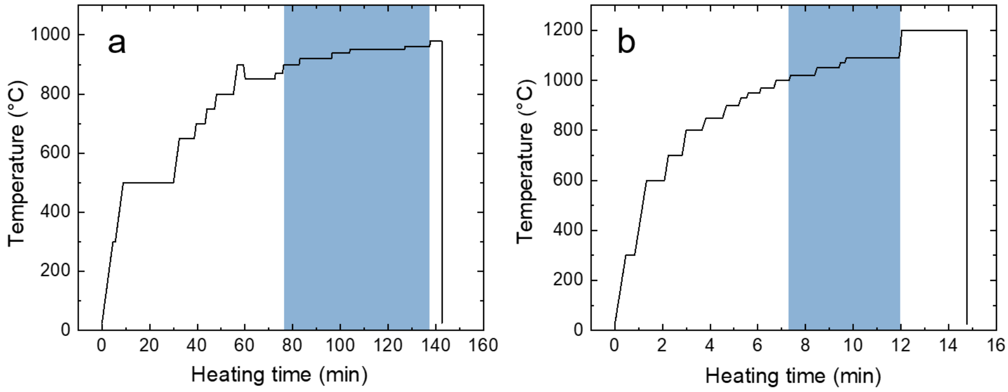

Figure S5. Plots of temperature versus heating time for the GaN film (a) and the nanowires (b). The blue areas represent the observation windows during the heating process.
